# Protective ventilation reduces *Pseudomonas aeruginosa* growth in lung tissue in a porcine pneumonia model

**DOI:** 10.1186/s40635-017-0152-3

**Published:** 2017-08-31

**Authors:** Jesper Sperber, Axel Nyberg, Miklos Lipcsey, Åsa Melhus, Anders Larsson, Jan Sjölin, Markus Castegren

**Affiliations:** 1 Centre for Clinical Research Sörmland, Uppsala University, Uppsala, Sweden; 2 Hedenstierna laboratory, Department of Surgical Sciences, Anesthesiology and Intensive Care, Uppsala University, Uppsala, Sweden; 3 Department of Medical Sciences, Section of Clinical Microbiology, Uppsala University, Uppsala, Sweden; 4 Department of Medical Sciences, Biochemical structure and function, Uppsala University, Uppsala, Sweden; 5 Department of Medical Sciences, Infectious Diseases, Uppsala University, Uppsala, Sweden; 6 Perioperative Medicine and Intensive Care, Karolinska University Hospital and CLINTEC, Karolinska Institute, Stockholm, Sweden; 7 Centre for Clinical Research Sörmland, Department of Anesthesiology & Intensive Care Mälarsjukhuset, SE-631 88 Eskilstuna, Sweden

**Keywords:** Critical care, Bacterial infections, Ventilators, mechanical, Protective ventilation, Models, animal

## Abstract

**Background:**

Mechanical ventilation with positive end expiratory pressure and low tidal volume, i.e. protective ventilation, is recommended in patients with acute respiratory distress syndrome. However, the effect of protective ventilation on bacterial growth during early pneumonia in non-injured lungs is not extensively studied. The main objectives were to compare two different ventilator settings on *Pseudomonas aeruginosa* growth in lung tissue and the development of lung injury.

**Methods:**

A porcine model of severe pneumonia was used. The protective group (*n* = 10) had an end expiratory pressure of 10 cm H_2_O and a tidal volume of 6 ml x kg^−1^. The control group (*n* = 10) had an end expiratory pressure of 5 cm H_2_O and a tidal volume of 10 ml x kg^−1^. 10^11^ colony forming units of *Pseudomonas aeruginosa* were inoculated intra-tracheally at baseline, after which the experiment continued for 6 h. Two animals from each group received only saline, and served as sham animals. Lung tissue samples from each animal were used for bacterial cultures and wet-to-dry weight ratio measurements.

**Results:**

The protective group displayed lower numbers of *Pseudomonas aeruginosa* (*p* < 0.05) in the lung tissue, and a lower wet-to-dry ratio (*p* < 0.01) than the control group. The control group deteriorated in arterial oxygen tension/inspired oxygen fraction, whereas the protective group was unchanged (*p* < 0.01).

**Conclusions:**

In early phase pneumonia, protective ventilation with lower tidal volume and higher end expiratory pressure has the potential to reduce the pulmonary bacterial burden and the development of lung injury.

**Electronic supplementary material:**

The online version of this article (10.1186/s40635-017-0152-3) contains supplementary material, which is available to authorized users.

## Background

Mechanical ventilation (MV) inherently carries the risks of cyclic atelectasis and over distension of alveoli, events that add to a pro-inflammatory response [1]. Protective ventilation, i.e. the avoidance of iatrogenic harm by the use of higher than traditional positive end expiratory pressure (PEEP) levels and smaller than traditional tidal volumes (V_T_), has an established role in the care of patients with acute respiratory distress syndrome (ARDS) [2]. Currently, some authors argue in favor of implementing protective ventilation to patient categories other than those suffering from ARDS in an attempt to prevent rather than to treat manifest lung injury [3]. In the current study we have compared two different ventilator settings that are considered safe under healthy lung conditions, and commonly found in clinical use in operating theaters [4]. These settings were previously compared during experimental endotoxinemia in pigs and proved to influence both systemic and organ-specific inflammation [5, 6]. We hypothesized that the benefits of protective ventilation seen in relation to inflammation would entail effects on bacterial counts in lung tissue in an experimental pneumonia setting. The aims of the study were to quantify how protective ventilation, before the onset of lung injury in healthy animals, affects the bacterial growth in lung tissue and the development of lung injury. We used a porcine pneumonia model with the bacterium *Pseudomonas (P.) aeruginosa*.

## Methods

Anesthesia, surgical procedure, protocol and physiologic measurements have been described in a previous publication [5], and a full account is provided in the supplementary files (Methods Supplement).

### Anesthesia and surgical procedure

**Fig. 1:**
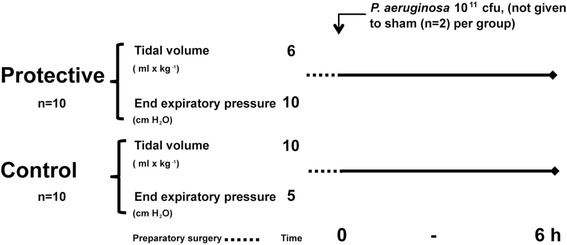
Overview of the experimental design. Ventilator settings during the whole experiment including preparations. Tracheostomy, mechanical ventilation, surgical preparations and preparation of bacteria underwent for approximately one hour (dashed line) in all animals. The inoculation of *P. aeruginosa* 10^11^ colony forming units (cfu) indicated the start of the experimental protocol at 0 h

The pigs were kept under general anesthesia in a supine position throughout the experiment. The time from induction of anesthesia until the experimental start point was approximately one hour in all animals and included the preparatory surgery and the preparation of the bacterial inoculum (Fig. 1).

The surgery contained a tracheostomy, a right-sided 5-French (F) arterial catheter, a central venous catheter, a Swan-Ganz pulmonary artery catheter, a 5F portal vein catheter via laparotomy and cannulation of the splenic hilus, and cystotomia catheter. In the current experiment the portal vein catheter was placed primarily to harmonize the surgical stimulus with the preceding experiments [5, 6]. To reduce the risk of bacterial contamination of the model cefuroxime 750 mg was administered after the surgical preparations. Before the start of the study protocol, a suction catheter was inserted blindly into the tracheal tube until it reached mechanical resistance. After a bronchoalevolar lavage (BAL) for culture and laboratory analyses the bacterial suspension was injected blindly into the lungs via the same catheter. A recruitment maneuver was performed with a plateau pressure of 30 cm H_2_O for 10 seconds (s). The inoculation of *P. aeruginosa* denoted the start of the experiment, i.e. baseline at 0 h.

### Bacterial inoculum

Two animals in each group served as sham animals and were not challenged with bacteria (Fig. 1). The aim of the bacterial preparation was to produce a 20 mL bolus consisting of 10^11^ colony forming units (cfu) of *P. aeruginosa* (5 × 10^9^ cfu x mL^−1^). The strain was isolated from a previous porcine experiment and naturally resistant to cefuroxime. It was O-antigen serotyped to O3 by a slide agglutination test with commercial antisera (Bio-Rad Laboratories AB, Solna, Sweden) at the section for Clinical Microbiology and Infectious Medicine (Uppsala, Sweden). Bacteria from over night cultures on Cystine-Lactose-Electrolyte-Deficient (CLED) agar (BD Diagnostics, Stockholm, Sweden) were dissolved in lysogeny broth (LB) according to Miller [7] (VWR, Leuven, Belgium). The optical density of the bacterial solution was measured with light absorbance spectrophotometry at a wavelength of 595 nm; a target value of 0.7 was reached by either dilution of the suspension or addition of more bacteria. One hundred mL of the final suspension were further diluted with another 100 mL of LB and incubated at 37 °C for 60 minutes (min). The incubated solution was centrifuged for 10 min at 20 °C to form bacterial pellets that were dissolved in 20 mL of sodium chloride 0.9%. One hundred μL were diluted 1:10^7^ to confirm the bacterial concentration of the bolus dose by culture on CLED agar.

### Protocol

The initial respirator settings (Servo i, Siemens Elema, Stockholm, Sweden) were in the protective group (*n* = 10): V_T_ 6 mL x kg^−1^, PEEP 10 cmH_2_O and respiratory rate (RR) 35 breaths x min^−1^; and in the control group (*n* = 10) V_T_ 10 mL x kg^−1^, PEEP 5 cmH_2_O and RR 25 breaths x min^−1^. Initial inspired fraction of oxygen (FiO_2_) was 0.3 for all animals, and adjustments were made to keep arterial partial pressure of oxygen (PaO_2_) between 10 and 18 kPa. Adjustments in ventilatory frequency were made to keep arterial partial pressure of carbon dioxide (PaCO_2_) between 4.5 and 6.5 kPa. During the early part of the experiment norepinephrine was used in a 40 μg bolus dose to treat falling mean arterial pressure (MAP) and rising mean pulmonary arterial pressure (MPAP) [8]. MAP below 60 mmHg during the later stages of the experiment was treated with Ringer’s acetate bolus doses of 15 mL x kg^−1^ and norepinephrine infusion of 20 μg x mL^−1^ with a starting rate of 5 mL x h^−1^.

### Measurements

All baseline measurements and sampling procedures were performed before the instillation of bacteria at 0 h. Lung tissue bacterial cultures and weight measurements were based on three dorsal samples from the right lung cranial, middle and caudal lobes, as well as three corresponding level samples from the left lung. Approximately 1 gram (g) from each sample was used for bacterial cultures. Three mL of sodium chloride 0.9% were added, followed by 4 min of mechanical homogenization with a Stomacher 80 Biomaster (Seward, Worthing, UK). One hundred μL were sequentially diluted until 1:10^4^ and cultured in a single repetition on CLED agar plates over night at 37 °C. The numbers of cfus from the countable plates were converted to units x g^−1^ lung tissue. The remainders of the six samples, ranging from 10 to 40 g, from each animal were weighed directly and after drying for 12 h at 60 °C. Bronchoalveolar lavages, performed by method of blind bronchial sampling with 20 mL of saline 0.9%, for cultures and cytokine measurements were performed at 0 h and before the end of the experiment at 6 h.

### Statistics

**Fig. 2:**
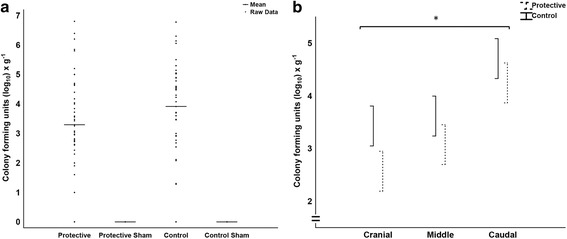
*P. aeruginosa* counts in lung tissue. **a** All *P. aeruginosa* counts from lung tissue samples in the experiment, log_10_ colony forming units per gram wet lung tissue, bars indicate the mean value in each group, sample number in Protective and Control were 48 each and in the sham groups 12 each. **b** *P. aeruginosa* counts at the three sample levels (cranial, middle, caudal) used in the general linear model analysis of the two main groups Protective and Control, log_10_ colony forming units per gram wet lung tissue, spreads are mean ± SE, the statistical test refers to group difference and not differences at individual levels, * denotes *p* < 0.05

The animals were allocated to treatment group by block randomization. Comparative group statistics were conducted on the animals challenged with bacteria (*n* = 16). Descriptive data for the sham animals not challenged with bacteria (*n* = 4) are presented in Figs. 2 and 4, and in a separate table (Table 5). The exclusion of the sham animals from comparative statistics did not lead to any altered significances in the results.

A general linear model (GLM) was used for group comparisons in the lung tissue sample variables (i.e. bacterial growth and wet-to-dry ratio) and in the repeated-measure variables. The main rationale for the choice of statistic method was that the six simultaneous lung tissue samples were dependent within each animal. Random effects were introduced into the model to account for the within-subject dependencies of the lung tissue samples and the repeated measures respectively [9]. The GLM equations were therefore mixes of fixed and random factors, i.e. mixed models.

The reason for not using six sample sites in the equation - but instead three levels (cranial, middle and caudal) each consisting of the right and left corresponding samples - was that we did not know if the inoculum was delivered to the right or left lung. Thus, we could not evaluate the spread of bacteria between the lungs, but it was possible to evaluate the caudal to cranial spread. The factors in the lung tissue variable equations were group (fixed), level (random) and group*level (random). The factors in the repeated measure variable equations were group (fixed) and group*time (random). The *p*-values and significances presented correspond to the group (fixed) factors and not the random or combined factors used in the equations.

Cytokines in plasma, inoculation dose and bacterial growth in lung tissue approximated a log-normal distribution and were logarithmically transformed before analysis. All BAL variables were of non-normal distribution and thus groups were compared with Mann-Whitney U tests. For data symmetry, the BAL cytokines were logarithmically transformed in the table presentation. A *p*-value of <0.05 was considered significant. Statistica™ (version 13, Statsoft, Tulsa, OK) was used in the statistical calculations and for the control of relevant assumptions. A senior statistician approved the statistical design.

No power calculation was conducted for this experiment since we had no previous data on bacterial growth in our models. The preceding experiments [5, 6, 10] utilized a power calculus based on a systemic TNF-alpha difference of 15% at 6 h, an alpha error of 0,05, a power of 0,8, and an SD of 10%, which has yielded six evaluable animals per group. Based on the preceding calculation and with the aim of reducing the number of animals and still allowing for a slightly larger variability in the bacterial outcomes we chose 8 animals per group in the current experiment.

## Results

Animal weights, the use of fluid boluses and norepinephrine according to protocol were similar between groups (Table 1).

**Table 1:** Weight, fluid, norepinephrine

	Protective (*n* = 10)	Control (*n* = 10)
Weight (kg)	24.6 ± 2.3	24.0 ± 2.8
Fluid bolus (15 mL x kg^−1^)	1	0
NE bolus 40 μg (n)	0	1
NE bolus 20 μg + infusion (n)	2	1

Descriptive and protocol data between Protective (*n* = 10) and Control (*n* = 10), sham animals (*n* = 2 per group) are included in the total, mean ± SD, (n) number, NE (norepinephrine), no comparative statistics were performed.

### Bacterial cultures and bronchoalveolar lavages

*P. aeruginosa* growth in lung tissue (cfu x g^−1^) was lower in the protective group than in the control group. Both groups displayed the highest bacterial counts at the caudal level and the lowest at the cranial level (Fig. 2).

Bacterial growth in BAL at 0 and 6 h did not differ between the groups. Both TNFα and IL6 levels in BAL were lower in the protective group at the start of the experimental protocol at 0 h, but the differences did not persist at the end of the experiment (6 h) (Table 2).

**Table 2:** Inoculation dose and bronchoalveolar lavages

Variable	Group	0 h	p	6 h	p
Inoculation dose	Protective (*n* = 8)	11.0 ± 0.1		-	
(log_10_cfu)	Control (*n* = 8)	10.9 ± 0.2	0.05	-	-
*P. aeruginosa* BAL	Protective (*n* = 8)	0.0(0.0/0.2)		4.0(2.9/4.8)	
(log_10_cfu × 100 μL^−1^)	Control (*n* = 8)	0.0(0.0/0.0)	N/A	3.8(3.3/4.6)	0.96
TNFα BAL	Protective (*n* = 8)	1.0(1.0/1.5)		3.6(3.1/3.7)	
(log_10_ng x L^−1^)	Control (*n* = 8)	1.8(1.7/1.9)	<0.05*	3.3(2.8/3.6)	0.37
IL6 BAL	Protective (*n* = 8)	1.7(1.7/1.7)		2.6(1.9/2.9)	
(log_10_ng x L^−1^)	Control (*n* = 8)	2.6(1.7/2.9)	<0.05*	2.6(2.0/3.0)	0.96

Inoculation dose of *Pseudomonas* (*P.*) *aeruginosa,* colony forming units (cfu), mean ± SD, *p*-value from general linear model. Growth in BAL (bronchoalveolar lavage), TNFα (tumor necrosis factor alpha) and IL6 (interleukin 6) in BAL, median(lower/upper quartile), *p*-values from Mann-Whitney U test, * denotes *p* < 0.05, N/A (not applicable).

Urea was measured *post-hoc* in plasma and BAL to calculate the dilution from saline to the alveolar lining fluid. However, most BAL samples had urea levels below the detection limit that made the dilution variable unusable. Urea data is presented only in the supplementary files (Data Supplement).

### Plasma cytokines and inflammatory cells

No differences were detected between the groups in plasma levels of TNFα, IL6, leukocytes or neutrophils (Table 3).

**Table 3:** Plasma cytokines and inflammatory cells

Variable	Group	0 h	3 h	6 h	p
TNFα	Protective (*n* = 8)	2.2 ± 0.4	2.5 ± 0.2	2.6 ± 0.2	
(log_10_ng x L^−1^)	Control (*n* = 8)	2.3 ± 0.3	2.5 ± 0.1	2.5 ± 0.1	0.71
IL6	Protective (*n* = 8)	2.0 ± 0.4	2.2 ± 0.4	3.2 ± 0.3	
(log_10_ng x L^−1^)	Control (*n* = 8)	1.9 ± 0.0	2.4 ± 0.3	3.1 ± 0.3	0.55
Leukocytes	Protective (*n* = 8)	16 ± 4	16 ± 4	16 ± 5	
(10^9^ x L^−1^)	Control (*n* = 8)	14 ± 5	15 ± 6	18 ± 7	0.58
Neutrophils	Protective (*n* = 8)	9 ± 4	8 ± 5	9 ± 5	
(10^9^ x L^−1^)	Control (*n* = 8)	7 ± 4	9 ± 5	11 ± 6	0.30

Cytokine levels in plasma for TNFα (tumor necrosis factor alpha), IL6 (interleukin 6), leukocytes and neutrophils, mean ± SD, *p*-values based on all measurements 0-6 h from the general linear model analysis. Additional data are presented in the supporting files (Additional file 1: Table S1).

### Lung injury, physiologic variables and hypoperfusion

**Fig. 3:**
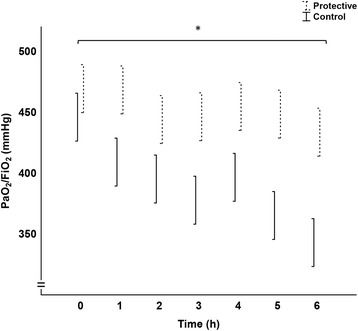
PaO_2_/FiO_2_ (mmHg). Arterial partial pressure of oxygen / inspired oxygen fraction, data from 0 to 6 h used in the general linear model analysis of the two main groups Protective and Control, spreads are mean ± SE, the statistical test refers to the group difference and not to differences at individual times, * denotes *p* < 0.01

**Fig. 4:**
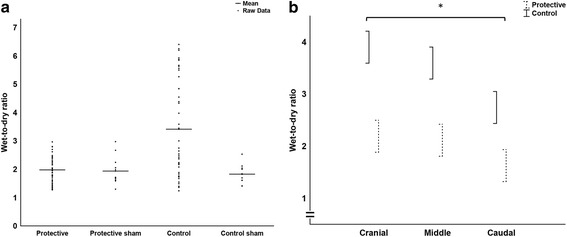
Wet-to-dry ratio. **a** All wet-to-dry ratios from lung tissue samples in the experiment, bars indicate the mean value in each group, sample number in Protective and Control were 48 each and in the sham groups 12 each. **b** Wet-to-dry ratios at the three sample levels (cranial, middle, caudal) used in the general linear model analysis of the two main groups Protective and Control, spreads are mean ± SE, the statistical test refers to group difference and not differences at individual levels, * denotes *p* < 0.01

The PaO_2_/FiO_2_ was higher and the wet-to-dry ratio lower in the protective group than in the control group (Figs. 3 and 4). Albumin was measured *post-hoc* in plasma and BAL for investigating alveolo-capillary permeability. The BAL samples were almost without exception below the detection limit, and hence this lung damage variable could not be used. Albumin data is presented only in the supplementary files (Data Supplement).

Peak airway pressure did not differ between the groups, whereas mean airway pressure was higher in the protective group and plateau pressure was higher in the control group. CI, MAP and MPAP were lower in the protective group, but heart rate and pulmonary capillary wedge pressure (PCWP) displayed no group differences. The temperature was lower in the protective group. There were no group differences in lactate levels in blood (Table 4).

**Table 4:** Physiologic variables and hypoperfusion

Variable	Group	0 h	3 h	6 h	p
P peak	Protective (*n* = 8)	19 ± 2	21 ± 3	22 ± 3	
(cmH_2_O)	Control (*n* = 8)	18 ± 2	20 ± 2	21 ± 2	0.35
P mean	Protective (*n* = 8)	13 ± 1	13 ± 1	13 ± 2	
(cmH_2_O)	Control (*n* = 8)	9 ± 1	9 ± 1	9 ± 1	<0.01*
P plateau	Protective (*n* = 8)	18 ± 2	19 ± 2	20 ± 3	
(cmH_2_O)	Control (*n* = 8)	17 ± 2	20 ± 2	21 ± 2	<0.05*
CI	Protective (*n* = 8)	2.6 ± 0.6	2.0 ± 0.2	2.1 ± 0.5	
(L x min^−1^ x m^−2^)	Control (*n* = 8)	3.2 ± 0.7	2.5 ± 0.3	2.4 ± 0.8	<0.05*
MAP	Protective (*n* = 8)	80 ± 16	68 ± 9	69 ± 14	
(mmHg)	Control (*n* = 8)	93 ± 22	79 ± 15	75 ± 20	<0.05*
MPAP	Protective (*n* = 8)	21 ± 2	23 ± 3	27 ± 5	
(mmHg)	Control (*n* = 8)	22 ± 4	29 ± 14	34 ± 14	<0.05*
HR	Protective (*n* = 8)	105 ± 19	84 ± 15	88 ± 16	
(beats x min^−1^)	Control (*n* = 8)	103 ± 20	94 ± 9	111 ± 12	0.06
PCWP	Protective (*n* = 8)	10 ± 3	9 ± 1	9 ± 2	
(mmHg)	Control (*n* = 8)	9 ± 3	9 ± 4	9 ± 4	0.93
Temperature	Protective (*n* = 8)	38.3 ± 0.6	37.8 ± 0.5	37.8 ± 0.8	
(°C)	Control (*n* = 8)	38.4 ± 0.9	38.6 ± 0.9	39.1 ± 1.0	<0.01*
Lactate	Protective (*n* = 8)	1.6 ± 0.5	1.3 ± 0.3	1.1 ± 0.2	
(mmol x L^−1^)	Control (*n* = 8)	1.8 ± 0.4	1.6 ± 0.7	1.5 ± 0.6	0.18

P (airway pressure in ventilator), CI (cardiac index), MAP (mean arterial pressure), MPAP (mean pulmonary arterial pressure), HR (heart rate), PCWP (pulmonary capillary wedge pressure), mean ± SD, *p*-values based on all measurements 0-6 h from the general linear model analysis, * denotes *p* < 0.05. Additional data are presented in the supporting files (Additional file 2: Table S2).

### Sham animals

Descriptive data for all variables from the sham animals (*n* = 4, two per ventilation group) that were not challenged with bacteria are presented without comparative statistics (Table 5).

**Table 5:** Sham animals

Variable	Group	0 h	3 h	6 h
*P. aeruginosa* lung	PS (*n* = 2)	-	-	0.0 ± 0.0
(log_10_cfu x g^−1^)	CS (*n* = 2)	-	-	0.0 ± 0.0
*P. aeruginosa* BAL	PS (*n* = 2)	0.0(0.0/0.0)	-	0.0(0.0/0.0)
(log_10_cfu × 100 μL^−1^)	CS (*n* = 2)	0.0(0.0/0.0)	-	0.0(0.0/0.0)
TNFα BAL	PS (*n* = 2)	1.5(1.5/1.5)	-	1.6(1.3/1.8)
(log_10_ng x L^−1^)	CS (*n* = 2)	1.4(1.0/1.7)	-	1.4(1.0/1.8)
IL6 BAL	PS (*n* = 2)	1.7(1.7/1.7)	-	1.7(1.7/1.7)
(log_10_ng x L^−1^)	CS (*n* = 2)	1.7(1.7/1.7)	-	1.7(1.7/1.7)
TNFα	PS (*n* = 2)	2.1 ± 0.1	2.2 ± 0.1	2.3 ± 0.1
(log_10_ng x L^−1^)	CS (*n* = 2)	2.0 ± 0.5	2.2 ± 0.0	2.1 ± 0.1
IL6	PS (*n* = 2)	-	1.9 ± 0.1	2.0 ± 0.2
(log_10_ng x L^−1^)	CS (*n* = 2)	1.6 ± 0.0	2.0 ± 0.0	1.7 ± 0.3
Leukocytes	PS (*n* = 2)	17 ± 9	20 ± 5	19 ± 6
(10^9^ x L^−1^)	CS (*n* = 2)	19 ± 8	21 ± 7	22 ± 7
Neutrophils	PS (*n* = 2)	10 ± 7	11 ± 3	9 ± 3
(10^9^ x L^−1^)	CS (*n* = 2)	7 ± 0	10 ± 0	11 ± 0
PaO_2_/FiO_2_	PS (*n* = 2)	454 ± 5	404 ± 2	448 ± 21
(mmHg)	CS (*n* = 2)	429 ± 34	366 ± 12	360 ± 11
Wet-to-dry ratio	PS (*n* = 2)	-	-	1.9 ± 0.5
	CS (*n* = 2)	-	-	1.8 ± 0.3
P peak	PS (*n* = 2)	18 ± 0	19 ± 0	19 ± 0
(cmH_2_O)	CS (*n* = 2)	15 ± 2	16 ± 4	17 ± 6
P mean	PS (*n* = 2)	12 ± 0	13 ± 1	13 ± 1
(cmH_2_O)	CS (*n* = 2)	8 ± 1	8 ± 1	9 ± 3
P plateau	PS (*n* = 2)	16 ± 0	17 ± 1	16 ± 0
(cmH_2_O)	CS (*n* = 2)	14 ± 2	15 ± 4	16 ± 7
CI	PS (*n* = 2)	2.5 ± 0.1	3.7 ± 0.7	3.2 ± 1.1
(L x min^−1^ x m^−2^)	CS (*n* = 2)	3.3 ± 0.3	3.2 ± 0.5	3.6 ± 1.1
MAP	PS (*n* = 2)	70 ± 1	81 ± 6	74 ± 14
(mmHg)	CS (*n* = 2)	84 ± 14	86 ± 4	81 ± 8
MPAP	PS (*n* = 2)	18 ± 2	21 ± 1	21 ± 3
(mmHg)	CS (*n* = 2)	18 ± 1	21 ± 1	21 ± 1
HR	PS (*n* = 2)	112 ± 6	103 ± 8	102 ± 23
(beats x min^−1^)	CS (*n* = 2)	100 ± 21	103 ± 10	113 ± 8
PCWP	PS (*n* = 2)	9 ± 1	7 ± 0	7 ± 1
(mmHg)	CS (*n* = 2)	8 ± 1	9 ± 0	8 ± 0
Temperature	PS (*n* = 2)	39.0 ± 0.2	37.8 ± 0.6	37.7 ± 1.5
(°C)	CS (*n* = 2)	39.3 ± 0.5	39.8 ± 1.4	40.3 ± 1.1
Lactate	PS (*n* = 2)	2.0 ± 0.4	0.9 ± 0.2	0.9 ± 0.1
(mmol x L^−1^)	CS (*n* = 2)	2.1 ± 0.5	1.0 ± 0.1	0.9 ± 0.1

Descriptive data for the two sham animals (not challenged with bacteria) from each group for all variables in the experiment, mean ± SD and median(lower/upper quartile). PS (protective sham), CS (control sham), *P*. (*Pseudomonas*), TNFα (tumor necrosis factor alpha), BAL (bronchoalveolar lavage), IL6 (interleukin 6), PaO_2_/FiO_2_ (arterial partial pressure of oxygen / inspired oxygen fraction), P (airway pressure in ventilator), CI (cardiac index), MAP (mean arterial pressure), MPAP (mean pulmonary arterial pressure), HR (heart rate), PCWP (pulmonary capillary wedge pressure). Additional data are presented in the supporting files (Additional file 3: Table S3).

## Discussion

We have shown that protective ventilation, consisting of medium low V_T_ of 6 ml x kg^−1^ and medium high PEEP of 10 cm H_2_O, affects the bacterial burden in the lungs during experimental pneumonia. *P. aeruginosa* counts in lung tissue were reduced and the development of lung injury was attenuated in the protectively ventilated animals. In bronchoalveolar lavages neither bacterial counts nor cytokines differed between the two groups.

Protective ventilation can potentially affect *P. aeruginosa* growth in lung tissue in multiple ways. Previously, the reduction of atelectasis formation by the appliance of PEEP was correlated to greater bacterial clearance in porcine pneumonia models [11, 12]. PEEP can, as demonstrated in rats, effectually stabilize a localized lung infection by preventing the dispersion of alveolar fluid from a primary site to different parts of the lungs [13, 14]. A PEEP-induced stabilization of the inoculate may have been present in the current experiment where the largest difference between the groups was found in the cranial segments of the lungs furthest away from the caudal bacterial deposition point. Elevated PEEP levels during high V_T_ ventilation have proved to reduce trans-alveolar albumin flux resulting in less lung tissue edema [13, 15]. Regrettably, our efforts to analyze trans-alveolar flux in the current experiment met with values below detection in BAL making the variable unusable. However, the protectively ventilated animals clearly displayed less edema than the control animals as seen in the wet-to-dry ratio variable. The edema difference may to an unknown extent have influenced the cellular response from alveolar macrophages (AM) to a *P. aeruginosa* challenge. The AM, a major effector of early resistance to bacterial infection, can be affected by ventilation [16]. In rats, edema formation was correlated to bacterial viability and to levels of clearance in vivo, and to impaired bactericidal activity of AM in vitro [17]. In a dog pneumonia model, using *P. aeruginosa* and comparing zero PEEP (ZEEP) to a PEEP of 10 cmH_2_O, there were quantitatively less bacteria and less lung damage in the PEEP animals after 24 h [18]. The bacteriologic result was later explained in a subsequent experiment by a relative dysfunction of the AM in the ZEEP lungs [19]. Very large tidal volumes undoubtedly produce lung damage and edema [15], but even moderate tidal volumes such as 10 ml x kg^−1^ can mediate lung deterioration when an inflammatory stimulus is present [20]. It has been postulated that mechanical ventilation, especially without PEEP and with larger V_T_, primarily induces surfactant dysfunction that precipitates atelectasis, edema formation, inflammation and ARDS [21]. One potential mechanism whereby the protective settings in the current experiment exerted its effects would be to preserve surfactant function during the early pneumonia phase, and thereby halting deterioration of lung physiological and immunological function. Further, the combined increase in PEEP and reduction in V_T_ in the protectively ventilated animals resulted in a higher mean airway pressure and a marginally lower plateau pressure. Driving pressure reduction (P plateau – PEEP), such as exemplified in the protective group, is one proposed central mediator of the lung protective effects seen in large ARDS trials [22]. These listed potential mechanistic explanations for the results were not addressed in the current study and would require different experimental layouts. They will remain to be addressed specifically in future experiments.

In regard to diagnosing pneumonia and quantifying bacteria, BAL is arguably inferior to lung tissue biopsies in all ways except applicability [23]. Contrary to the lung tissue cultures, *P. aeruginosa* counts in BAL did not separate the groups in the current experiment. We believe the discrepancy to depend on the unspecific BAL sampling method generating too much variability between the individuals.

There were differences in the inflammatory cytokines TNFα and IL6 in BAL before the bacterial inoculation at 0 h, at which point the animals had been under group-separating MV for approximately one hour during the surgical preparation. In no other inflammatory marker in the experiment (i.e. TNFα and IL6 in plasma, leukocytes, neutrophils and temperature) could a difference in 0 h values be found between the protective and control groups. Only a few animals in the control group displayed elevated BAL cytokine values at 0 h. To rule out the possibility that these animals affected the bacterial outcomes of the study, *post-hoc* recalculations without them were performed. These recalculations did not change the statistical significance of the difference in bacterial growth. In a preceding experiment TNFα levels in plasma rose rapidly during the preparatory surgery for two hours, whereas IL6 levels only rose after the surgery and peaked three hours later [5]. The same difference in reaction time was shown in another endotoxin porcine model where TNFα peaked at one and IL6 at three hours respectively after the inflammatory stimulus [24]. Although it is possible that the ventilator settings in the current experiment made a difference in both TNFα and IL6 in such a short time as one hour during surgery, it is more likely a chance result from the BAL method. The BAL cytokines did not differ significantly at the end of the experiment, which contrasts to the marked difference in edema development in the lungs. We made *post-hoc* measurements of urea in plasma and BAL to calculate a dilution factor for each BAL sample made from the sampling saline to the alveolar lining fluid [25]. Most BAL samples were too diluted to allow detection of urea even though the sample volumes were increased, hence dilution factors could not be calculated. Adverse ventilation has in rat models been correlated to edema formation and TNFα production [26, 27], whereas low V_T_ ventilation has been correlated to the opposite picture [28]. It is possible or even likely that an inflammatory cytokine difference was present in the lung tissue but went undetected with our sampling method. The higher temperature in the control animals could be interpreted as a sign of a more pronounced inflammatory reaction to the infection, albeit not reflected in systemic cytokines in the current experiment. The circulation was more hypodynamic in the protectively ventilated animals. The lower CI was possibly due to a combination of a relatively lower systemic inflammatory response with lower fever and heart rate, and to a PEEP induced negative effect on the right ventricle as indicated by the lower MAP [29]. However, neither group manifested hypoperfusion as indicated by the low levels of lactate in systemic blood.

We recognize limitations of our study design. The short experiment with direct bacterial inoculation was not designed to resemble the clinical pathology of a ventilator-associated pneumonia [30], but rather to examine the impact of early mechanical ventilation on an early-stage lung infection. In addition, the experiment was not a lung injury model. The control group displayed a higher degree of edema and a clear decline in PaO_2_/FiO_2_, but never reached the level defining ARDS within the experimental period [31]. As lungs display progressive heterogeneity during worsening of lung injury protective ventilation could have varying effects if applied early or late in the course of pneumonia [32]. The results would therefore only possibly extrapolate to clinical benefits in later stage pneumonia or in more injured lungs. The limited inflammatory strength of the model may have allowed the lungs to contain the infection compartmentalized, which could account for the lack of group differences in inflammatory markers on the systemic level. However, a pneumonia model without large systemic effects might have increased the possibility to study specific pulmonary effects of the ventilator intervention. The major benefits and translational relevance of the study are that it was conducted in a large animal model, and that the ventilator settings in both groups are commonly used clinically and are still largely, but arguably, considered non-harmful in healthy lungs [4, 33].

## Conclusions

In early phase pneumonia, protective ventilation with lower tidal volume and higher end expiratory pressure has the potential to reduce the pulmonary bacterial burden and the development of lung injury.

## Additional files

Additional file 1: Table S1. Plasma cytokines and inflammatory cells. Cytokine levels in arterial plasma for TNFα (tumor necrosis factor alpha), IL6 (interleukin 6), leukocytes and neutrophils, mean ± SD, *p*-values based on all measurements 0-6 h from the general linear model analysis. (DOC 42 kb)

Additional file 2: Table S2. Physiologic variables and hypoperfusion. P (airway pressure in ventilator), CI (cardiac index), MAP (mean arterial pressure), MPAP (mean pulmonary arterial pressure), HR (heart rate), PCWP (pulmonary capillary wedge pressure), mean ± SD, *p*-values based on all measurements 0-6 h from the general linear model analysis, * denotes *p* < 0.05. (DOC 56 kb)

Additional file 3: Table S3. Sham animals. Descriptive data for the two sham animals (not challenged with bacteria) from each group for all variables in the experiment, mean ± SD and median(lower/upper quartile). PS (protective sham), CS (control sham), *P*. (*Pseudomonas*), TNFα (tumor necrosis factor alpha), BAL (bronchoalveolar lavage), IL6 (interleukin 6), PaO_2_/FiO_2_ (arterial partial pressure of oxygen / inspired oxygen fraction), P (airway pressure in ventilator), CI (cardiac index), MAP (mean arterial pressure), MPAP (mean pulmonary arterial pressure), HR (heart rate), PCWP (pulmonary capillary wedge pressure). (DOC 83 kb)

## Abbreviations

AM: alveolar macrophage
ARDS: acute respiratory distress syndrome
BAL: bronchoalveolar lavage
C: Celsius
cfu: colony forming unit
CI: cardiac index
CLED: Cystine-Lactose-Electrolyte-Deficient
cm: centimeter
CV: coefficient of variation
ELISA: enzyme-linked immunosorbent assay
F: French
FiO_2_: inspired fraction of O_2_
g: gram
GLM: general linear model
h: hour
H_2_O: water
IL6: interleukin 6
kg: kilogram
kPa: kilo Pascal
LB: Lysogeny broth
MAP: mean arterial pressure
mg: milligram
min: minute
mL: milliliter
MPAP: mean pulmonary arterial pressure
MV: mechanical ventilation
n: number
P: pressure
*P.*: *Pseudomonas*
PaO_2_: arterial partial pressure of oxygen
PCWP: pulmonary capillary wedge pressure
PEEP: positive end expiratory pressure
s: second
TNFα: tumor necrosis factor alpha
V_T_: tidal volume
ZEEP: zero end expiratory pressure
μg: microgram
