# Supplementary material for: Protective ventilation reduces Pseudomonas aeruginosa growth in lung tissue in a porcine pneumonia model
Source: Intensive Care Med Exp. 2017 Aug 31;5:40. doi: 10.1186/s40635-017-0152-3 (PMC5578946; doi:10.1186/s40635-017-0152-3)
Supplement: Supplementary file 1 — Plasma cytokines and inflammatory cells. Cytokine levels in arterial plasma for TNFα (tumor necrosis factor alpha), IL6 (interleukin 6), leukocytes and neutrophils, mean ± SD, p-values based on all measurements 0-6 h from the general linear model analysis. (DOC 42 kb) [file 40635_2017_152_MOESM1_ESM.doc]

**Table Supplement 1**

| **Variable** | **Group** | **0 h** | **1 h** | **2 h** | **3 h** | **4 h** | **5 h** | **6 h** | **p** |
| --- | --- | --- | --- | --- | --- | --- | --- | --- | --- |
| **TNFα** | Protective (n=8) | 2.2±0.4 | 2.4±0.3 | 2.4±0.2 | 2.5±0.2 | 2.6±0.2 | 2.6±0.2 | 2.6±02 |  |
| (log_10_ng x L^-1^) | Control (n=8) | 2.3±0.3 | 2.5±0.2 | 2.5±0.1 | 2.5±0.1 | 2.6±0.1 | 2.6±0.1 | 2.5±0.1 | 0.71 |
| **IL6** | Protective (n=8) | 2.0±0.4 | 2.1±0.4 | 2.0±0.5 | 2.2±0.4 | 2.5±0.3 | 2.9±0.3 | 3.2±0.3 |  |
| (log_10_ng x L^-1^) | Control (n=8) | 1.9±0.0 | 2.2±0.3 | 2.2±0.3 | 2.4±0.3 | 2.8±0.2 | 3.1±0.2 | 3.1±0.3 | 0.55 |
| **Leukocytes** | Protective (n=8) | 16±4 | 14±3 | 14±4 | 16±4 | 16±3 | 16±4 | 16±5 |  |
| (10^9^ x L^-1^) | Control (n=8) | 14±5 | 12±8 | 16±8 | 15±6 | 15±6 | 15±7 | 18±7 | 0.58 |
| **Neutrophils** | Protective (n=8) | 9±4 | 7±4 | 7±5 | 8±5 | 9±4 | 9±5 | 9±5 |  |
| (10^9^ x L^-1^) | Control (n=8) | 7±4 | 6±6 | 9±6 | 9±5 | 9±5 | 10±5 | 11±6 | 0.30 |
|  |  |  |  |  |  |  |  |  |  |
|  |  |  |  |  |  |  |  |  |  |
|  |  |  |  |  |  |  |  |  |  |
|  |  |  |  |  |  |  |  |  |  |
|  |  |  |  |  |  |  |  |  |  |
|  |  |  |  |  |  |  |  |  |  |

**Table Supplement 1. Plasma cytokines and inflammatory cells**

Cytokine levels in arterial plasma for TNFα (tumor necrosis factor alpha), IL6 (interleukin 6), leukocytes and neutrophils, mean±SD, p-values based on all measurements 0-6 h from the general linear model analysis.
