# Supplementary material for: Protective ventilation reduces Pseudomonas aeruginosa growth in lung tissue in a porcine pneumonia model
Source: Intensive Care Med Exp. 2017 Aug 31;5:40. doi: 10.1186/s40635-017-0152-3 (PMC5578946; doi:10.1186/s40635-017-0152-3)
Supplement: Supplementary file 2 — Physiologic variables and hypoperfusion. P (airway pressure in ventilator), CI (cardiac index), MAP (mean arterial pressure), MPAP (mean pulmonary arterial pressure), HR (heart rate), PCWP (pulmonary capillary wedge pressure), mean ± SD, p-values based on all measurements 0-6 h from the general linear model analysis, * denotes p < 0.05. (DOC 56 kb) [file 40635_2017_152_MOESM2_ESM.doc]

**Table Supplement 2**

| **Variable** | **Group** | **0 h** | **1 h** | **2 h** | **3 h** | **4 h** | **5 h** | **6 h** | **p** |
| --- | --- | --- | --- | --- | --- | --- | --- | --- | --- |
|  |  |  |  |  |  |  |  |  |  |
| **P peak** | Protective (n=8) | 19±2 | 20±2 | 20±2 | 21±3 | 21±2 | 21±3 | 22±3 |  |
| (cmH_2_O) | Control (n=8) | 18±2 | 19±2 | 20±2 | 20±2 | 21±2 | 21±2 | 21±2 | 0.35 |
| **P mean** | Protective (n=8) | 13±1 | 12±1 | 12±1 | 13±1 | 13±1 | 13±1 | 13±2 |  |
| (cmH_2_O) | Control (n=8) | 9±1 | 9±1 | 9±1 | 9±1 | 9±1 | 9±1 | 9±1 | <0.01* |
| **P plateau** | Protective (n=8) | 18±2 | 17±2 | 18±2 | 19±2 | 18±2 | 19±2 | 20±3 |  |
| (cmH_2_O) | Control (n=8) | 17±2 | 19±2 | 20±2 | 20±2 | 21±2 | 20±2 | 21±2 | <0.05* |
| **CI** | Protective (n=8) | 2.6±6 | 2.6±0.4 | 2.1±0.4 | 2.0±0.2 | 2.2±0.3 | 2.1±0.6 | 2.1±0.5 |  |
| (L x min^-1^ x m^-2^) | Control (n=8) | 3.2±0.7 | 2.4±0.4 | 2.3±0.3 | 2.5±0.3 | 2.6±0.5 | 2.6±0.6 | 2.4±0.8 | <0.05* |
| **MAP** | Protective (n=8) | 80±16 | 71±10 | 72±11 | 68±9 | 68±9 | 68±13 | 69±14 |  |
| (mmHg) | Control (n=8) | 93±22 | 80±12 | 77±13 | 79±15 | 70±28 | 80±16 | 75±20 | <0.05* |
| **MPAP** | Protective (n=8) | 21±2 | 22±2 | 23±2 | 23±3 | 24±4 | 25±5 | 27±5 |  |
| (mmHg) | Control (n=8) | 22±4 | 26±10 | 28±13 | 29±14 | 31±13 | 33±13 | 34±14 | <0.05* |
| **HR** | Protective (n=8) | 105±19 | 98±20 | 84±17 | 84±15 | 89±14 | 88±14 | 88±16 |  |
| (beats x min^-1^) | Control (n=8) | 103±20 | 94±18 | 89±15 | 94±9 | 103±9 | 107±8 | 111±12 | 0.06 |
| **PCWP** | Protective (n=8) | 10±3 | 9±3 | 8±2 | 9±1 | 8±2 | 8±2 | 9±2 |  |
| (mmHg) | Control (n=8) | 9±3 | 9±4 | 8±4 | 9±4 | 9±4 | 9±4 | 9±4 | 0.93 |
| **Temperature** | Protective (n=8) | 38.3±0.6 | 37.9±0.6 | 37.8±0.5 | 37.8±0.5 | 37.8±0.6 | 37.8±0.6 | 37.8±0.8 |  |
| (°C) | Control (n=8) | 38.4±0.9 | 38.3±1.0 | 38.5±0.9 | 38.6±0.9 | 38.8±1.0 | 39.0±0.9 | 39.1±1.0 | <0.01* |
| **Lactate artery** | Protective (n=8) | 1.6±0.5 | - | - | 1.3±0.3 | - | - | 1.1±0.2 |  |
| (mmol x L^-1^) | Control (n=8) | 1.8±0.4 | - | - | 1.6±0.7 | - | - | 1.5±0.6 | 0.18 |
| **Lactate portal vein** | Protective (n=8) | 1.7±0.5 | - | - | 1.3±0.4 | - | - | 1.2±0.4 |  |
| (mmol x L^-1^) | Control (n=8) | 2.0±0.6 | - | - | 1.6±0.6 | - | - | 1.6±0.6 | 0.14 |

**Table Supplement 2. Physiologic variables and hypoperfusion**

P (airway pressure in ventilator), CI (cardiac index), MAP (mean arterial pressure), MPAP (mean pulmonary arterial pressure), HR (heart rate), PCWP (pulmonary capillary wedge pressure), mean±SD, p-values based on all measurements 0-6 h from the general linear model analysis, * denotes p<0.05.
