# Supplementary material for: Protective ventilation reduces Pseudomonas aeruginosa growth in lung tissue in a porcine pneumonia model
Source: Intensive Care Med Exp. 2017 Aug 31;5:40. doi: 10.1186/s40635-017-0152-3 (PMC5578946; doi:10.1186/s40635-017-0152-3)
Supplement: Supplementary file 3 — Sham animals. Descriptive data for the two sham animals (not challenged with bacteria) from each group for all variables in the experiment, mean ± SD and median(lower/upper quartile). PS (protective sham), CS (control sham), P. (Pseudomonas), TNFα (tumor necrosis factor alpha), BAL (bronchoalveolar lavage), IL6 (interleukin 6), PaO2/FiO2 (arterial partial pressure of oxygen / inspired oxygen fraction), P (airway pressure in ventilator), CI (cardiac index), MAP (mean arterial pressure), MPAP (mean pulmonary arterial pressure), HR (heart rate), PCWP (pulmonary capillary wedge pressure). (DOC 83 kb) [file 40635_2017_152_MOESM3_ESM.doc]

**Table Supplement 3**

| **Variable** | **Group** | **0 h** | **1 h** | **2 h** | **3 h** | **4 h** | **5 h** | **6 h** |
| --- | --- | --- | --- | --- | --- | --- | --- | --- |
| ***P. aeruginosa* lung** | PS (n=2) | - | - | - | - | - | - | 0.0±0.0 |
| (log_10_ cfu x g^-1^) | CS (n=2) | - | - | - | - | - | - | 0.0±0.0 |
| ***P. aeruginosa* BAL** | PS (n=2) | 0.0(0.0/0.0) | - | - | - | - | - | 0.0(0.0/0.0) |
| (log_10_ cfu x 100µL^-1^) | CS (n=2) | 0.0(0.0/0.0) | - | - | - | - | - | 0.0(0.0/0.0) |
| **TNFα BAL** | PS (n=2) | 1.5(1.5/1.5) | - | - | - | - | - | 1.6(1.3/1.8) |
| (log_10_ng x L^-1^) | CS (n=2) | 1.4(1.0/1.7) | - | - | - | - | - | 1.4(1.0/1.8) |
| **IL6 BAL** | PS (n=2) | 1.7(1.7/1.7) | - | - | - | - | - | 1.7(1.7/1.7) |
| (log_10_ng x L^-1^) | CS (n=2) | 1.7(1.7/1.7) | - | - | - | - | - | 1.7(1.7/1.7) |
| **TNFα** | PS (n=2) | 2.1±0.1 | 2.2±0.1 | 2.3±0.0 | 2.2±0.1 | 2.3±0.0 | 2.3±0.1 | 2.3±0.1 |
| (log_10_ng x L^-1^) | CS (n=2) | 2.0±0.5 | 2.3±0.0 | 2.3±0.0 | 2.2±0.0 | 2.2±0.1 | 2.1±0.0 | 2.1±0.1 |
| **IL6** | PS (n=2) | - | - | 1.5±0.1 | 1.9±0.1 | 1.8±0.2 | 1.9±0.2 | 2.0±0.2 |
| (log_10_ng x L^-1^) | CS (n=2) | 1.6±0.0 | 2.0±0.0 | 2.1±0.0 | 2.0±0.0 | 2.1±0.0 | 2.1±0.0 | 1.7±0.3 |
| **Leukocytes** | PS (n=2) | 17±9 | 17±6 | 17±8 | 20±5 | 20±6 | 19±7 | 19±6 |
| (10^9^ x L^-1^) | CS (n=2) | 19±8 | 14±6 | 18±9 | 21±7 | 21±8 | 21±8 | 22±7 |
| **Neutrophils** | PS (n=2) | 10±7 | 9±4 | 9±4 | 11±3 | 11±3 | 10±4 | 9±3 |
| (10^9^ x L^-1^) | CS (n=2) | 7±0 | 5±0 | 6±0 | 10±0 | 9±0 | 10±0 | 11±0 |
| **PaO_2_/FiO_2_** | PS (n=2) | 454±5 | 448±43 | 434±5 | 404±2 | 466±9 | 472±27 | 448±21 |
| (mmHg) | CS (n=2) | 429±34 | 410±21 | 385±11 | 366±12 | 365±4 | 366±12 | 360±11 |
| **Wet to dry ratio** | PS (n=2) | - | - | - | - | - | - | 1.9±0.5 |
|  | CS (n=2) | - | - | - | - | - | - | 1.8±0.3 |
| **P peak** | PS (n=2) | 18±0 | 18±0 | 19±0 | 19±0 | 19±0 | 20±1 | 19±0 |
| (cmH_2_O) | CS (n=2) | 15±2 | 16±2 | 15±3 | 16±4 | 16±5 | 16±4 | 17±6 |
| **P mean** | PS (n=2) | 12±0 | 12±0 | 13±1 | 13±1 | 13±1 | 13±1 | 13±1 |
| (cmH_2_O) | CS (n=2) | 8±1 | 8±1 | 8±1 | 8±1 | 8±1 | 8±1 | 9±3 |
| **P plateau** | PS (n=2) | 16±0 | 16±0 | 17±1 | 17±1 | 16±0 | 17±1 | 16±0 |
| (cmH_2_O) | CS (n=2) | 14±2 | 15±2 | 14±4 | 15±4 | 15±5 | 15±6 | 16±7 |
| **CI** | PS (n=2) | 2.5±0.1 | 3.8±0.1 | 3.2±0.3 | 3.7±0.7 | 3.3±0.9 | 3.0±1.1 | 3.2±1.1 |
| (L x min^-1^ x m^-2^) | CS (n=2) | 3.3±0.3 | 4.0±0.7 | 3.5±0.6 | 3.2±0.5 | 3.5±0.7 | 3.6±0.7 | 3.6±1.1 |
| **MAP** | PS (n=2) | 70±1 | 67±1 | 78±4 | 81±6 | 80±8 | 81±8 | 74±14 |
| (mmHg) | CS (n=2) | 84±14 | 92±8 | 94±11 | 86±4 | 80±1 | 83±4 | 81±8 |
| **MPAP** | PS (n=2) | 18±2 | 19±0 | 20±1 | 21±1 | 21±2 | 21±1 | 21±3 |
| (mmHg) | CS (n=2) | 18±1 | 22±1 | 20±1 | 21±1 | 21±0 | 22±2 | 21±1 |
| **HR** | PS (n=2) | 112±6 | 103±8 | 95±12 | 103±8 | 98±11 | 99±20 | 102±23 |
| (beats x min^-1^) | CS (n=2) | 100±21 | 100±1 | 103±1 | 103±10 | 110±11 | 113±13 | 113±8 |
| **PCWP** | PS (n=2) | 9±1 | 8±1 | 7±0 | 7±0 | 7±1 | 8±1 | 7±1 |
| (mmHg) | CS (n=2) | 8±1 | 8±1 | 9±1 | 9±0 | 8±0 | 8±0 | 8±0 |
| **Temperature** | PS (n=2) | 39.0±0.2 | 38.1±0.2 | 38.0±0.6 | 37.8±0.6 | 37.6±1.0 | 37.7±1.1 | 37.7±1.5 |
| (°C) | CS (n=2) | 39.3±0.5 | 38.9±0.6 | 39.4±1.2 | 39.8±1.4 | 40.1±1.2 | 40.2±1.1 | 40.3±1.1 |
| **Lactate (artery)** | PS (n=2) | 2.0±0.4 | - | - | 0.9±0.2 | - | - | 0.9±0.1 |
| (mmol x L^-1^) | CS (n=2) | 2.1±0.5 | - | - | 1.0±0.1 | - | - | 0.9±0.1 |
|  |  |  |  |  |  |  |  |  |
|  |  |  |  |  |  |  |  |  |
|  |  |  |  |  |  |  |  |  |
|  |  |  |  |  |  |  |  |  |
|  |  |  |  |  |  |  |  |  |
|  |  |  |  |  |  |  |  |  |
|  |  |  |  |  |  |  |  |  |
|  |  |  |  |  |  |  |  |  |

**Table Supplement 3.** **Sham animals**

Descriptive data for the two sham animals (not challenged with bacteria) from each group for all variables in the experiment, mean±SD and median(lower/upper quartile). PS (protective sham), CS (control sham), *P*. (*Pseudomonas*), TNFα (tumor necrosis factor alpha), BAL (bronchoalveolar lavage), IL6 (interleukin 6), PaO_2_/FiO_2_ (arterial oxygen tension / inspired oxygen fraction), P (airway pressure in ventilator), CI (cardiac index), MAP (mean arterial pressure), MPAP (mean pulmonary arterial pressure), HR (heart rate), PCWP (pulmonary capillary wedge pressure).
